# eHealth Interventions for Treatment and Prevention of Depression, Anxiety, and Insomnia During Pregnancy: Systematic Review and Meta-analysis

**DOI:** 10.2196/31116

**Published:** 2022-02-21

**Authors:** Katherine A Silang, Pooja R Sohal, Katherine S Bright, Jennifer Leason, Leslie Roos, Catherine Lebel, Gerald F Giesbrecht, Lianne M Tomfohr-Madsen

**Affiliations:** 1 Department of Psychology University of Calgary Calgary, AB Canada; 2 Department of Community Health Sciences Cumming School of Medicine University of Calgary Calgary, AB Canada; 3 Women’s Mental Health Clinic Foothills Medical Centre Alberta Health Services Calgary, AB Canada; 4 Department of Anthropology and Archaeology University of Calgary Calgary, AB Canada; 5 Department of Psychology and Pediatrics University of Manitoba Winnipeg, MB Canada; 6 Children’s Hospital Research Institute of Manitoba Winnipeg, MB Canada; 7 Department of Radiology Alberta Children’s Hospital Research Institute Hotchkiss Brain Institute Calgary, AB Canada; 8 Alberta Children’s Hospital Research Institute Calgary, AB Canada; 9 Department of Pediatrics University of Calgary Calgary, AB Canada

**Keywords:** eHealth, pregnancy, depression, anxiety, insomnia, mobile phone

## Abstract

**Background:**

Pregnancy is associated with an increased risk for depression, anxiety, and insomnia. eHealth interventions provide a promising and accessible treatment alternative to face-to-face interventions.

**Objective:**

The objective of this systematic review and meta-analysis is to determine the effectiveness of eHealth interventions in preventing and treating depression, anxiety, and insomnia during pregnancy. Secondary aims are to identify demographic and intervention moderators of effectiveness.

**Methods:**

A total of 5 databases (PsycINFO, Medline, CINAHL, Embase, and Cochrane) were searched from inception to May 2021. Terms related to eHealth, pregnancy, randomized controlled trials (RCTs), depression, anxiety, and insomnia were included. RCTs and pilot RCTs were included if they reported an eHealth intervention for the prevention or treatment of depression, anxiety, or insomnia in pregnant women. Study screening, data extractions, and quality assessment were conducted independently by 2 reviewers from an 8-member research team (KAS, PRS, Hangsel Sanguino, Roshni Sohail, Jasleen Kaur, Songyang (Mark) Jin, Makayla Freeman, and Beatrice Valmana). Random-effects meta-analyses of pooled effect sizes were conducted to determine the effect of eHealth interventions on prenatal mental health. Meta-regression analyses were conducted to identify potential moderators.

**Results:**

In total, 17 studies were included in this review that assessed changes in depression (11/17, 65%), anxiety (10/17, 59%), and insomnia (3/17, 18%). Several studies included both depression and anxiety symptoms as outcomes (7/17, 41%). The results indicated that during pregnancy, eHealth interventions showed small effect sizes for preventing and treating symptoms of anxiety and depression and a moderate effect size for treating symptoms of insomnia. With the exception of intervention type for the outcome of depressive symptoms, where mindfulness interventions outperformed other intervention types, no significant moderators were detected.

**Conclusions:**

eHealth interventions are an accessible and promising resource for treating symptoms of anxiety, depression, and insomnia during pregnancy. However, more research is necessary to identify ways to increase the efficacy of eHealth interventions for this population.

**Trial Registration:**

PROSPERO (International Prospective Register of Systematic Reviews) CRD42020205954; https://www.crd.york.ac.uk/prospero/display_record.php?RecordID=205954

## Introduction

### Background

Meta-analyses show high rates of depression [1] and anxiety disorders [2] during pregnancy. Sleep problems are also common during pregnancy; they increase as the pregnancy progresses and are often comorbid with other mental health problems [3-6]. Untreated antenatal mental health problems are associated with an increased risk for poor birth outcomes, such as miscarriage [7], preterm birth [2,8], and low birth weight [2,8]. Similarly, poor sleep during pregnancy is a predictor of poor birth outcomes [9], such as shorter gestational age in addition to increased risk for developing postpartum depression [6].

Symptoms of antenatal depression, anxiety, and insomnia, if left untreated, can persist long into the postpartum period as many symptoms postpartum begin antenatally [6,10,11]. Furthermore, the effects of psychological distress during pregnancy can have long-lasting developmental [12,13], emotional [14], behavioral [14], and cognitive impairments [14] on the child. Prenatal and postpartum maternal mental health concerns have been linked to altered brain structure in preschool children [15]. Together, these findings emphasize the importance of providing pregnant women with timely, accessible, and culturally safe interventions to better treat and support the mental health of all women or birth parents.

A confluence of evidence now shows that depression, anxiety, and insomnia can be effectively treated using in-person individual or group psychotherapy for the perinatal period [16,17]. On the basis of this evidence and the clear harm of untreated antenatal mental health problems, public health and medical agencies around the world are recommending routine screening and treatment for depression and anxiety during pregnancy [18-20]. Studies have found that women who are screened for depression during pregnancy, as opposed to the postpartum period, are more likely to follow up with treatment [21], which can lead to the prevention of further adverse outcomes [17].

Despite the strong arguments for antenatal screening and treatment of mental health problems, resources for the treatment of these concerns are limited, leaving mental health needs unrecognized and contributing to fetal risk along with persistent postpartum mental health problems [22]. Owing to the high rates of mental health and sleep problems during pregnancy, limited screening, and limited treatment resources, researchers and hospital administrators are increasingly looking to eHealth as a way to address unmet needs [23,24]. Screening alone, even without further treatment, has shown significant reductions in depression during the perinatal period [25]. Even in the presence of simple and effective screening tools, it is estimated that health care professionals detect only 25% of women with postpartum depression and even less with other perinatal mental health disorders [26] and up to 70% of pregnant or postpartum women will fail to seek treatment [27]. As a result, only 15% of women with a perinatal mental health disorder will receive evidence-based care [28] and these rates are lower in marginalized groups [29] and in fathers and partners [30].

eHealth is a new area in health care that focuses on the delivery of health services and information through web-based programs, remote monitoring, teleconsultation, and mobile device–supported care [31]. eHealth’s accessible nature aids in providing treatment to rural or remote areas, where patients otherwise would not have access to treatment and can involve lower intensity and more cost-effective delivery of services than in-person intervention, meaning that it may reach a larger number of patients [32].

The relevance of the use of eHealth interventions has only increased given that the COVID-19 pandemic has heightened psychological distress and sleep problems around the world. Pregnancy is already a period of vulnerability for mental health concerns [33-36] and a recent rapid review and meta-analysis of depression and anxiety during pregnancy during the COVID-19 pandemic reported that rates of depression and anxiety in pregnant women across the world are elevated compared with prepandemic levels [37]. The prevalence of insomnia has also increased during the COVID-19 pandemic [38], including during pregnancy. In addition to the need to investigate anxiety and depressive symptoms, insomnia is also important to be investigated as it is considered to be a transdiagnostic mechanism for various mental health concerns [39]. The elevation of mental health concerns during pregnancy has highlighted the need for accessible and timely solutions.

Although many eHealth interventions already exist, such as mobile health app for smartphones, very few are evidence-based [40,41]. Moreover, meta-analyses evaluating the effectiveness of eHealth interventions demonstrate mixed findings [42]. For example, prevention and treatment effects for depression appear to be small and dropout rates are high when there is no human monitoring and mood feedback. In contrast, moderate to large effect sizes are seen for eHealth interventions for insomnia [43,44]. The field of eHealth in the context of pregnancy is relatively new. Consequently, a systematic review and meta-analysis are needed to determine the effectiveness of eHealth interventions during pregnancy for the treatment of depression, anxiety, and insomnia symptoms.

### Aims of the Study

This study is a systematic review and meta-analysis of the data from available randomized controlled trials (RCTs) published to date on eHealth interventions delivered during pregnancy to prevent or treat depression, anxiety, and insomnia. Meta-estimates are conducted separately for each outcome (anxiety, depression, and insomnia). A secondary goal of the review is to identify moderators of treatment effects. Potential moderators investigated include frequency of treatment (less frequent or more frequent), method of eHealth delivery (SMS text messaging, app, internet, and computer), treatment provider (health care provider, researcher, and self), risk of bias, type of control group (active and nonactive), treatment goal (treatment and prevention), baseline mental health symptoms (above or below clinical threshold), number of sessions, structure of the intervention (guided or unguided), and intervention type (hybrid or asynchronous). To clarify further, hybrid eHealth interventions refer to eHealth interventions where a component of the intervention was completed in person, if there was in-person contact. Asynchronous interventions refer to eHealth interventions, which were delivered completely via the web.

## Methods

### Search Strategy

A total of 5 electronic databases (ie, CINAHL with full text, PsycINFO, Medline or PubMed, Cochrane CENTRAL, and Embase) were searched using key terms to capture eHealth or digital interventions, RCTs, depression, anxiety, and insomnia during pregnancy to retrieve all relevant peer-reviewed articles from 1957 to May 2021. Subject headings were used in databases when appropriate. No filters or limits were applied to ensure that no articles were missed. Recognized articles were exported to a web-based systematic review program, Covidence (Veritas Health Innovation) [45] and duplicates were removed. This investigation followed the methods outlined by the Cochrane Collaboration Handbook [46] and the standards set by PRISMA (Preferred Reporting Items for Systematic Review and Meta-Analysis) [47,48]. The remaining articles were reviewed for inclusion using Covidence [45]. The study was registered with PROSPERO through the University of York Center for Reviews and Dissemination (CRD42020205186). The full search is available in Multimedia Appendix 1.

### Study Selection

The initial abstract review was calibrated to ensure that the interrater reliability was >85%. Abstract eligibility was determined independently by each reviewer for all the identified articles. Conflicts were resolved by consensus with the first (KS) and second (PS) authors along with the research assistants (Roshni Sohail, Jasleen Kaur, Beatrice Valmana, Hangsel Sanguino, Makayla Freeman, and Songyang (Mark) Jin). Reference lists of the included articles and related review articles were inspected for any missed or relevant articles.

### Inclusion and Exclusion Criteria

**Figure 1:**
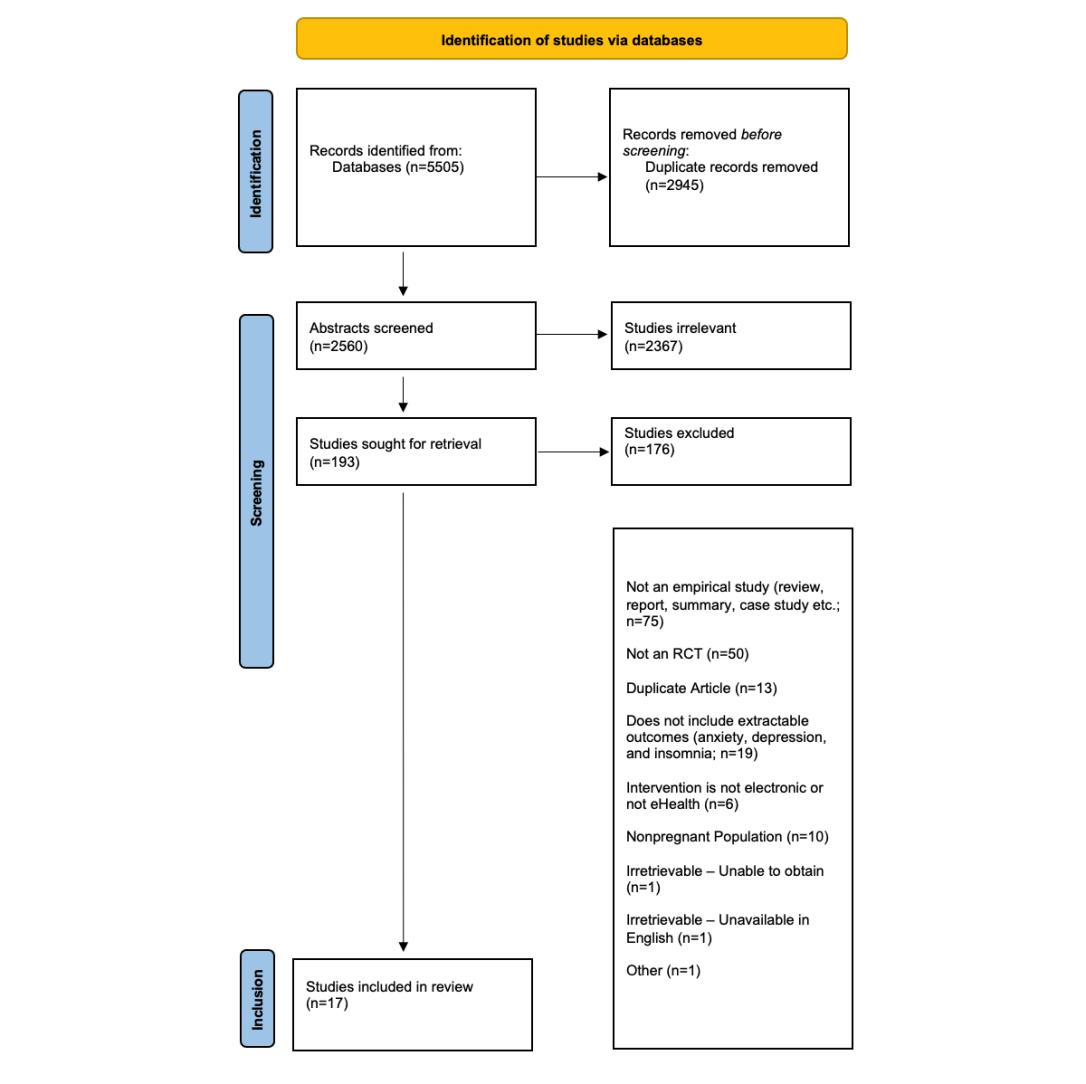
PRISMA (Preferred Reporting Items for Systematic Review and Meta-Analysis) flow chart. RCT: randomized controlled trial.

To be included in the review, studies had to be written in English and evaluate an eHealth intervention for anxiety, depression, or insomnia. eHealth interventions were defined and restricted to interventions that were delivered in an electronic capacity (eg, video therapy sessions, telephone, SMS text messaging, self-help interventions, and recorded therapy sessions). Studies had to be an RCT by study design. The intervention was required to occur before labor; however, the assessment of outcomes could occur in the postpartum period. Studies were excluded if (1) were not an RCT, (2) they did not have a control group, (3) they included a nonpregnant sample, (4) the interventions were not delivered electronically, (5) they were review articles, (6) they were case reports, (7) they used a previously included sample, or (8) they did not include continuous scores on a symptom measure of depression, anxiety, or insomnia. A flow chart of article inclusion and exclusion is shown in Figure 1.

### Data Extraction

The remaining articles were divided and extracted independently by 2 reviewers from an 8-member research team (KAS, PRS, Hangsel Sanguino, Roshni Sohail, Jasleen Kaur, Songyang (Mark) Jin, Makayla Freeman, and Beatrice Valmana). Conflicts were resolved by consensus with the coders and the first and second authors. Extracted data included authors' names; publication year; country in which the research was conducted; sample demographics; pregnancy characteristics; intervention characteristics; administration details; and assessment information of depression, anxiety, or insomnia for all groups. Additional sample characteristics that were extracted when possible included sample size, age, gestational age during intervention baseline, ethnicity and race, sex, and gender breakdown of participants within the invention. The name of the intervention (when applicable), description, method of administration, degree of interaction and guidance from the provider (if applicable) during the intervention, and time spent by participants on the intervention were extracted. Information about depression, anxiety, and insomnia outcomes extracted included rates or effect sizes of all groups postintervention. Authors of included articles were contacted for additional information if studies had missing or incomplete data that precluded them from the analyses. If author contact was unsuccessful (ie, the author did not respond to an email request or no longer had access to data), the studies were excluded from the full-text review.

### Data Analysis

Meta-analyses were conducted using the Comprehensive Meta-Analysis Software [49]. Sample sizes for each group (control and intervention), along with means and SDs of mental health symptoms for all study groups following the intervention (postintervention assessments, follow-ups, etc) were used to calculate meta-estimates of levels of depression, anxiety, or insomnia postintervention using random-effects meta-analyses. The overall meta-analysis computed a pooled Hedges *g* effect size, along with 95% CIs, for eHealth interventions across all included studies. A Hedges *g* of 0.20, 0.50, and 0.80 can be interpreted as small, moderate, and large effect sizes, respectively [50]. Stratified analyses were conducted according to the outcome (anxiety, depression, and insomnia). Separate meta-regression analyses with random-effects models were conducted when there were enough studies (3/17, 18%) that included at least one of the moderators of interest.

### Quality Assessment

To assess the quality of the studies, the Cochrane Collaboration’s Risk of Bias Tool was used. The tool assesses seven criteria common to RCTs (random sequence generation, allocation concealment, selective reporting, blinding of participants and personnel, blinding of outcome assessment, incomplete outcome data, and other sources of bias) in which bias could occur. Quality indicators from the studies were extracted by 2 reviewers. Discrepancies in quality indicator scores were resolved by the first author (KS). Total scores ranged from 0 to 7, with higher scores indicating a greater risk of bias.

## Results

### Study Selection

The search returned 5505 results, which were reduced to 2560 (46.50%) after duplicates were removed. Of the 2560 articles, 2367 (92.46%) articles were excluded after reviewing the titles and abstracts. At the full-text level, 7.54% (193/2560) of the articles were retrieved. From these 193 articles, 23 (11.9%) were included for extraction and, of them, 17 (89%) were included in this review. In all, 3 authors were contacted for additional information; 2 of whom replied and were included in the review. No additional articles were retrieved from additional searches of the reference lists. The article screening process is detailed in Figure 1.

### Characteristics of Included Studies

Full details of each study are presented in Table 1. Mean participant age ranged from 25.97 (SD 6.01) to 37.80 (SD 2.31) years and 100% of the participants were women. None of the included studies assessed fathers. Mean gestational age ranged from 15.9 (SD 6.3) to >30 weeks at the start of the trial. Of the 71% (12/17) of the studies where data about ethnicity were reported, 10 (83%) studies included 50-80% of participants who identified as White [51-60] and 6 (50%) studies had >80% of the total sample identified as White [51-56].

No studies required a formal diagnosis of anxiety, depression, or insomnia at baseline. Studies primarily delivered the eHealth intervention through the computer or internet (12/17, 71% of the included studies) [51-57,59-61,65,66]. Of these 12 studies, 4 (33%) were delivered via a mobile app on a smartphone [58,62-64] and 1 (8%) was delivered through SMS text messages [67]. It should be noted that although telephone-based studies were also eHealth interventions of interest, the review did not identify any telephone-based studies. Regarding the frequency at which the intervention was delivered, 12% (2/17) of the studies were considered to be low frequency (defined as an intervention that was accessed once, twice, or monthly) [52,66] and 88% (15/17) of the studies were considered to be high frequency (defined as an intervention that was delivered weekly or daily) [51,53-65,67]. In considering who delivered the intervention, treatment providers varied, as 12% (2/17) of the studies used health care [62,64] providers to deliver the intervention, 24% (4/17) of the studies used researchers to deliver the intervention [51,65-67], and 65% (11/17) of the studies used other means [52-57,59-61,63]. *Other* was subjectively defined as an intervention that was self-administered, but indirectly provided or developed by another organization, researcher, or clinician.

Interventions used to treat or prevent depression included cognitive behavioral therapy (CBT; 3/17, 18%) [54,56,65], psychoeducation (4/17, 24%) [52,55,59,62], mindfulness (3/17, 18%) [53,63,64], and attention bias modification training (1/17, 6%) [58].

Interventions used to treat or prevent anxiety included CBT (2/17, 12%) [51,54], general education about the perinatal period (2/17, 12%) [66,67], psychoeducation (2/17, 12%) [52,62], mindfulness (3/17, 18%) [53,63,64], and attention bias modification training (1/17, 6%) [58]. CBT for insomnia (CBT-I) was the only type of intervention used to improve symptoms of insomnia.

Symptoms of anxiety, depression, or insomnia were assessed by using validated questionnaires. Of the 17 included studies, 10 (59%) studies measured anxiety symptoms, 11 (65%) studies measured depressive symptoms, and 3 (18%) studies measured insomnia symptoms.

Of the 59% (10/17) studies that measured anxiety, 1 (10%) study measured anxiety using the Spielberger Trait Anxiety Inventory [51], 2 (20%) studies used the Depression Anxiety Stress Scale–Anxiety [58,62], 1 (10%) study used the Hamilton Anxiety Rating Scale [58], 1 (10%) study used the Visual Analogue Scale for Anxiety [66], 1 (10%) study used the Hospital Anxiety Depression Scale–Anxiety [52], 4 (40%) studies used the Generalized Anxiety Disorder-7 [53,54,63,64], and 1 (10%) study used an unspecified anxiety measure [67] (Table 2).

**Table 1:** Characteristics of included studies (N=17).

Study; country	Name of intervention	Intervention, N; control, N	Age of sample (years), intervention, mean (SD); control, mean (SD)	Gestational age (weeks)^a^, intervention, mean (SD); control, mean (SD)	Type of control group	Type of eHealth	Type of intervention	Outcome
Cain et al [61]; United States	Go To Sleep! internet-based CBT-I^b^	27; 26	28.5 (5.8); 29.8 (5.3)	19.6 (3.6); 22.8 (2.6)	Waitlist condition	Internet	CBT	Insomnia
Felder et al [57]; United States	Sleepio: Big Health; digital CBT-I	105; 103	33.9 (3.38); 33.2 (4)	17.1 (6.4); 18.1 (6.3)	Psychoeducation	Internet	CBT	Insomnia
Kalmbach et al [60]; United States	Sleepio: Big Health; digital CBT-I	46; 45	28.91 (4.21); 29.16 (4.11)	N/A^c^	Psychoeducation	Internet	CBT	Insomnia
Heller et al [52]; Netherlands;	MamaKits Onling: internet-based intervention	79; 80	32.08 (4.61); 31.94 (4.83)	<30; <30	TAU^d^	Internet	Psychoeducation	Anxiety and depression
Chan et al [62]; China	iParent app	330; 330	31.3 (4.6); 31.2 (4.5)	N/A	TAU	Mobile app	Psychoeducation	Anxiety and depression
Duffecy et al [56]; United States	Sunnyside Cognitive Behavioral Therapy e-Intervention	17; 6	30.5 (4.05; total sample)	20-28	eHealth intervention of reduced intensity	Internet	CBT	Depression
Haga et al [55]; Norway	Mamma Mia: web-based program	678; 664	31.0 (4.6); 31.1 (4.5)	21-25	TAU	Internet	Psychoeducation	Depression
Yang et al [63]; China	WeChat (mobile) Messages: mobile app	62; 61	31.31 (4.87); 30.38 (3.91)	25.52 (1.84); 26.33 (3.45)	TAU	Mobile app	Mindfulness	Anxiety and depression
Krusche et al [53]; United Kingdom	Be Mindful: website	107; 78	32.7 (mode=34; total sample)	>12	Waitlist condition	Internet	Mindfulness	Anxiety and depression
Loughnan et al [54]; Australia	MUMentum Pregnancy program: internet-delivered CBT	36; 41	31.69 (4.44); 31.54 (3.63)	20.54 (6.01); 22.63 (5.76)	TAU	Internet	CBT	Anxiety and depression
Dennis-Tiwary et al [58]; United States	ABMT^e^	15; 14	34.67 (4.39); 31.14 (6.16)	22.44 (2.43); 20-28	Placebo condition	Mobile app	ABMT	Anxiety and depression
Sun et al [64]; China	WeChat (mobile) Messages; mobile app	84; 84	30.27 (3.80); 29.55 (4.21)	13.82 (2.0); 14.41 (2.2)	eHealth intervention of reduced intensity	Mobile app	Spirits Healing app and mindfulness	Anxiety and depression
Forsell et al [65]; Sweden	Internet-delivered CBT	22; 20	31.2 (3.7); 30.8 (5.3)	15.9 (6.5); 18.6 (6.5)	TAU	Internet	CBT	Depression
Scherer et al [51]; Switzerland	Internet-based cognitive behavioral stress management; internet	31; 27	32.90 (3.49); 31.11 (3.50)	28.32 (2.96); 29.11 (2.47)	Placebo condition	Internet	CBT	Anxiety
Barrera et al [59]; United States	Internet-based mood management intervention	57; 54	29.81 (6.09); 30.59 (4.99)	20.51 (10.37); 19.42 (10.42)	eHealth intervention of reduced intensity	Internet	Psychoeducation	Depression
Hanprasertpong et al [66]; Thailand	Computer-assisted instruction	157; 164	37.8 (2.31); 37.5 (2.62)	16-20; 16-20	Paper version of the intervention	Computer or internet	General education	Anxiety
Jareethum et al [67]; Thailand	SMS text messaging intervention	32; 29	28.72 (4.9); 25.97 (6.1)	<28; <28;	TAU	SMS text message	General education	Anxiety

^a^The deviation from *mean (SD)* format in few studies is owing to unavailability of data.

^b^CBT-I: cognitive behavioral therapy for insomnia.

^c^N/A: not applicable.

^d^TAU: treatment as usual.

^e^ABMT: attention bias modification training.

**Table 2:** Anxiety outcome measures used by each study (N=10).

Study	Anxiety measure						
	STAI^a^	DASS-A^b^	HAMA^c^	VAS^d^	HADS^e^	GAD-7^f^	Unspecified
Heller et al [52]					✓		
Chan et al [62]		✓					
Yang et al [63]						✓	
Krusche et al [53]						✓	
Loughnan et al [54]						✓	
Dennis-Tiwary et al [58]		✓	✓				
Sun et al [64]						✓	
Scherer et al [51]	✓						
Hanprasertpong et al [66]				✓			
Jareethum et al [67]							✓

^a^STAI: State Trait Anxiety Inventory.

^b^DASS-A: Depression Anxiety Stress Scale–Anxiety.

^c^HAMA: Hamilton Anxiety Rating Scale.

^d^VAS: Visual Analogue Scale.

^e^HADS: Hospital Anxiety Depression Scale.

^f^GAD-7: Generalized Anxiety Disorder–7.

For symptoms of depression, of the 65% (11/17) of the studies, 7 (64%) studies used the Edinburgh Postnatal Depression Scale [52-55,62,64,65], 4 (36%) studies used the Patient Health Questionnaire-9 [53,54,56,63], 2 (18%) studies used the Center for Epidemiologic Studies Depression Scale [52,59], 1 (9%) study used the Hamilton Depression Rating Scale [56], 1 (9%) study used the Inventory of Depression and Anxiety Scale [56], 1 (9%) study used the Depression Anxiety Stress Scale–Depression [58], 1 (9%) study used the Montgomery-Asberg Depression Rating Scale [65], and 1 (9%) study used the Work and Social Adjustment Scale–Depression [65] (Table 3). For symptoms of insomnia, all 3 (100%) studies used the Pittsburgh Sleep Quality Index and the Insomnia Severity Index [57,60,61] (Table 4).

**Table 3:** Depression outcome measures used by each study (N=11).

Study	Depression measure							
	EPDS^a^	PHQ-9^b^	CES-D^c^	HDRS^d^	IDAS^e^	DASS-D^d^	MADRS^g^	WSAS^h^
Heller et al [52]	✓		✓					
Chan et al [62]	✓							
Duffecy et al [56]		✓		✓	✓			
Haga et al [55]	✓							
Yang et al [63]		✓						
Krusche et al [53]	✓	✓						
Loughnan et al [54]	✓	✓						
Dennis-Tiwary et al [58]						✓		
Sun et al [64]	✓							
Forsell et al [65]	✓						✓	✓
Barrera et al [59]			✓					

^a^EPDS: Edinburgh Postnatal Depression Scale.

^b^PHQ-9: Patient Health Questionnaire-9.

^c^CES-D: Center for Epidemiologic Studies Depression Scale.

^d^HDRS: Hamilton Depression Rating Scale.

^e^IDAS: Inventory of Depression and Anxiety Scale.

^f^DASS-D: Depression Anxiety Stress Scale–Depression.

^g^MADRS: Montgomery-Asberg Depression Rating Scale.

^h^WSAS: Work and Social Adjustment Scale.

**Table 4:** Insomnia outcome measures used by each study (N=3).

Study	Insomnia measure	
	ISI^a^	PSQI^b^
Cain et al [61]	✓	✓
Felder et al [57]	✓	✓
Kalmbach et al [60]	✓	✓

^a^ISI: Insomnia Severity Index.

^b^PSQI: Pittsburgh Sleep Quality Index.

Of the 17 studies, 7 (41%) studies assessed both anxiety and depressive symptoms [52-54,58,62-64]. Most of the control conditions specified that control participants received treatment as usual (TAU) from their health care providers (7/17, 41%) [52,54,55,62,63,65,67]. Some studies defined their control group as a waitlist condition (2/17, 12%) [53,61], a placebo condition (2/17, 12%) [51,58], an eHealth intervention of reduced intensity (3/17, 18%) [56,59,64], a paper version of the intervention (1/17, 6%) [66], or psychoeducation (3/17, 18%) [57,60,63].

### Risk of Bias in the Included RCTs

The results of bias assessments are shown in Table 5. Risk was rated as low for the 17 studies that were included. The most common risk of bias was owing to other biases, which were not explicitly mentioned in the quality assessment tool (ie, sampling bias). In general, the risk of selection, reporting, and attrition biases were low. The presence of other biases was judged as high in 10 (59%) of the 17 studies. Of the 17 included studies, 15 (88%) were judged to have a high risk of bias in at least one domain.

**Table 5:** Outcomes from included studies (N=17).

Study	Outcome	Study quality rating	Hedges *g*	*P* value	Intervention effect
Cain et al [61]	Insomnia	3	0.576	.06	The study was marginally significant in reducing insomnia symptoms following the intervention for women in the CBT-I^a^ group.
Felder et al [57]	Insomnia	3	0.688	<.001	Results from the study found those who received digital CBT-I experienced significantly greater reductions in insomnia symptom severity compared with women in the control group.
Kalmbach et al [60]	Insomnia	1	0.403	.06	CBT-I patients reported lower insomnia symptoms on the ISI^b^ and PSQI^c^ after treatment than controls; however, this was marginally significant.
Heller et al [52]	Anxiety and depression	1	Anxiety: 0.076; depression: –0.10	Anxiety: .70; depression: .96	No significant differences were found between the intervention group and the control group for both anxiety and depression.
Chan et al [62]	Anxiety and depression	1	Anxiety: –0.045; depression: 0.219	Anxiety: .56; depression: .02	Scores of depression significantly decreased in the intervention group when compared with the control group; however, scores of anxiety did not significantly decrease when comparing the intervention group with the control group.
Duffecy et al [56]	Depression	3	0.696	.29	Study results participants in the web-based intervention had reduced scores of depression when compared with the control group; however, this was not significant.
Haga et al [55]	Depression	1	0.121	.03	At all 4 follow-up time points of this study, pregnant people participating in the *Mamma Mia* had significantly lower depressive scores in comparison with the control group.
Yang et al [63]	Anxiety and depression	2	Anxiety: 0.868; depression: 0.933	Anxiety: <.001; depression: <.001	In comparison with the control group who had received in-person treatment, the participants belonging to the WeChat intervention reported significant reduction in anxiety and depressive scores.
Krusche et al [53]	Anxiety and depression	1	Anxiety: 0.641; depression: 0.677	Anxiety: .02; depression: .01	There was a significant reduction in scores between intervention and waitlist groups, regarding anxiety and depressive symptoms.
Loughnan et al [54]	Anxiety and depression	0	Anxiety: 0.588; depression: 0.300	Anxiety: .01; depression: .19	The analysis indicates that the iCBT^d^ group demonstrated no significant group by time interactions for depression symptom reduction. However, the iCBT group showed significantly reduced anxiety symptoms.
Dennis-Tiwary et al [58]	Anxiety and depression	1	Anxiety: –0.305; depression: 0.068	Anxiety: .40; depression: .85	Results found that individuals in the ABMT^e^ group did not show significant improvements in anxiety and depression.
Sun et al [64]	Anxiety and depression	2	Anxiety: 0.182; depression: 0.155	Anxiety: .24; depression: .08	Mindfulness training participants reported a decreased risk of positive depressive symptoms and anxiety symptoms in comparison with controls; however, this was not significant.
Forsell et al [65]	Depression	1	0.739	.02	Depression symptoms significantly decreased in the intervention group compared with the control group.
Scherer et al [51]	Anxiety	0	0.096	.71	Levels of stress and anxiety did not significantly decrease in the intervention group when compared with the control group.
Barrera et al [59]	Depression	3	–0.425	.16	Following the intervention, depression scores in the intervention group did not statistically differ from the control group.
Hanprasertpong et al [66]	Anxiety	3	0.010	.93	Anxiety following the intervention was reduced significantly in both groups in comparison with baseline; however, no significant differences existed among groups after the intervention.
Jareethum et al [67]	Anxiety	3	0.624	.02	In comparison with the control group who received treatment as usual, women receiving SMS text messages during the antenatal period demonstrated significantly decreased levels of anxiety.

^a^CBT-I: cognitive behavioral therapy for insomnia.

^b^ISI: Insomnia Severity Index.

^c^PSQI: Pittsburgh Sleep Quality Index.

^d^iCBT: internet-based CBT.

^e^ABMT: attention bias modification training.

### Effectiveness of eHealth Interventions for Treatment of Depressive Symptoms During Pregnancy

#### Overview

**Figure 2:**
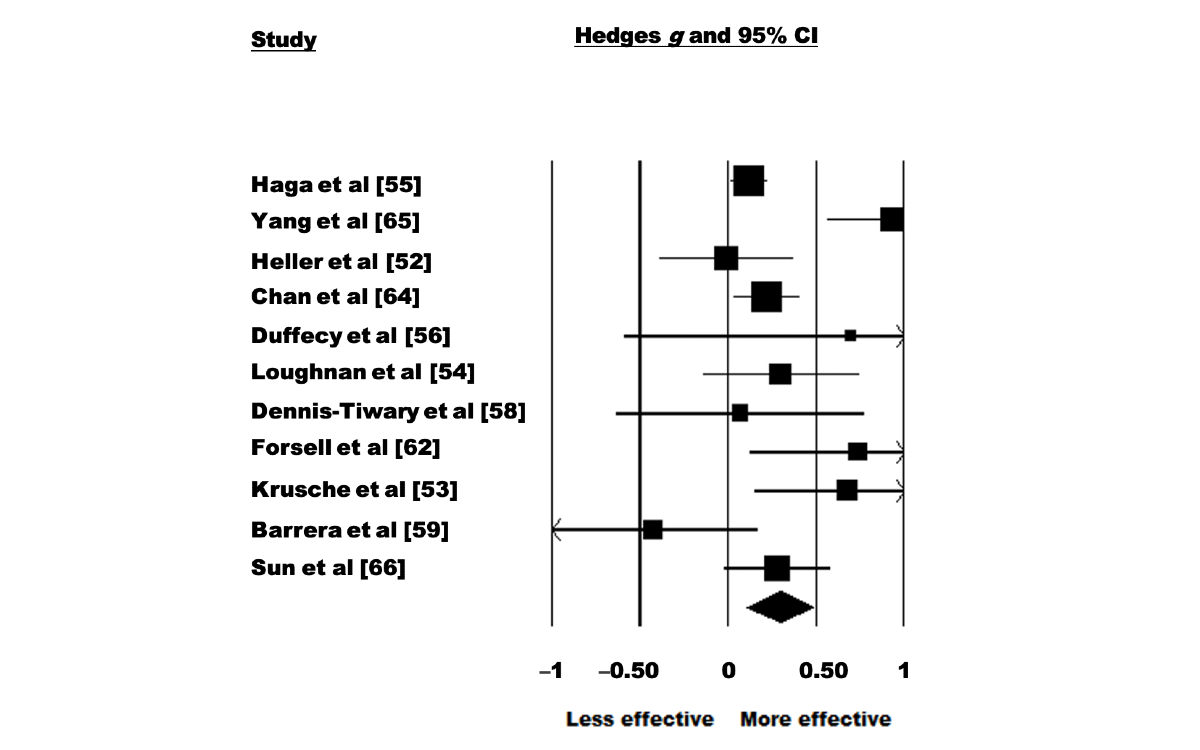
Depression–forest plot [52-56,58,59,62,64-66].

**Figure 3:**
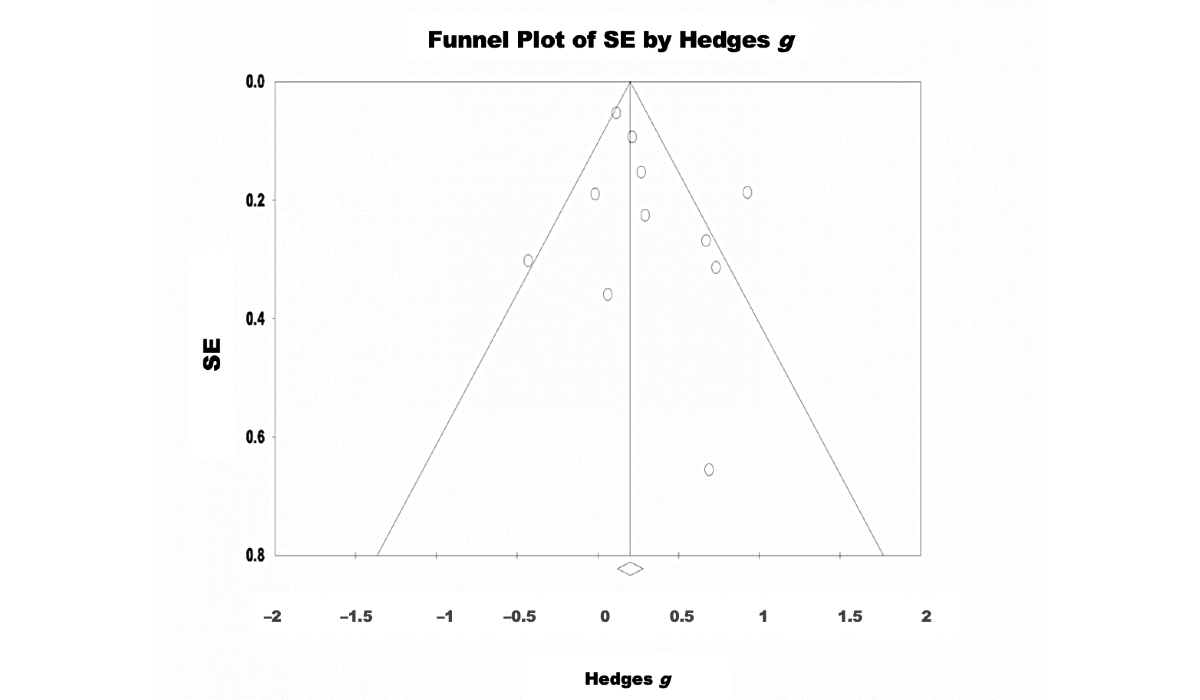
Depression–funnel plot.

A random-effects model was used to analyze the 65% (11/17) of the studies that assessed the effectiveness of eHealth interventions for the treatment of depressive symptoms. There were 2458 participants included in total (intervention: 1221, 49.67% and control: 1237, 50.32%). The pooled effect size reflected a significant effect of eHealth interventions on depressive symptoms with a small effect size (Hedges *g*=0.293, 95% CI 0.207-0.478; Z=3.090; *P=*.002; Figure 2). Significant heterogeneity was observed among the studies (Q=29.789; *P=*.001; *I*^2^=66.431). The test of asymmetry funnel plot is displayed in Figure 3. Egger regression test found no evidence of publication bias (b=1.02; *t*_9_=1.23; SE 0.831; *P*=.25). The Begg and Mazumdar rank correlation was nonsignificant (Kendall *S* statistic=9; *Τ*=0.163; Z=0.701; *P*=.48).

#### Sensitivity Analyses

After systematically removing one study at a time, it was observed that 64% (7/11) of the studies affected the meta-estimate of the effect size of eHealth intervention during pregnancy by >5% [52,53,55,58,59,62,63]. Of the 11 studies, 5 (45%) studies affected the meta-estimate such that it made the estimate larger, however, a significant association was still noted without the studies included (*P*<.001-.009) [52,55,58,59,62]. Of the 11 studies, 2 (18%) studies affected the meta-estimate such that they made the estimate smaller; however, a significant association was still noted when each individual study was excluded (*P=*.004-.006) [53,63].

### Effectiveness of eHealth Interventions for Treatment of Anxiety Symptoms During Pregnancy

#### Overview

**Figure 4:**
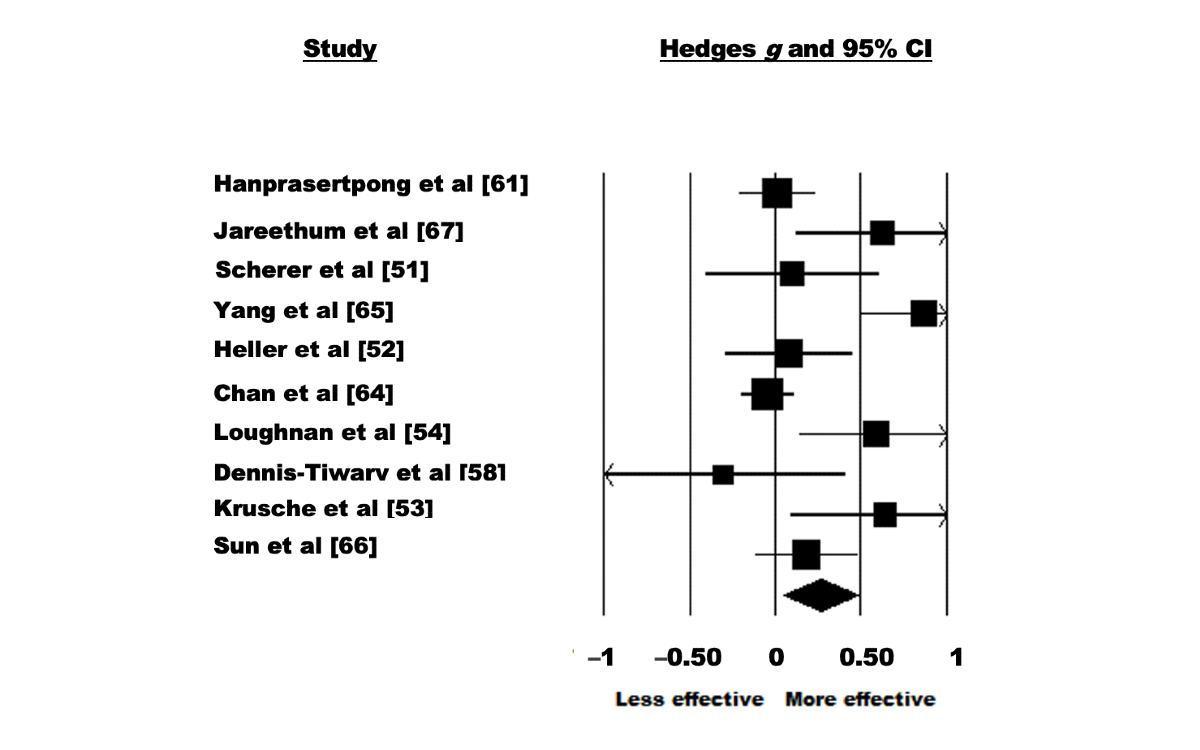
Anxiety–forest plot [51-54,58,61,64-67].

**Figure 5:**
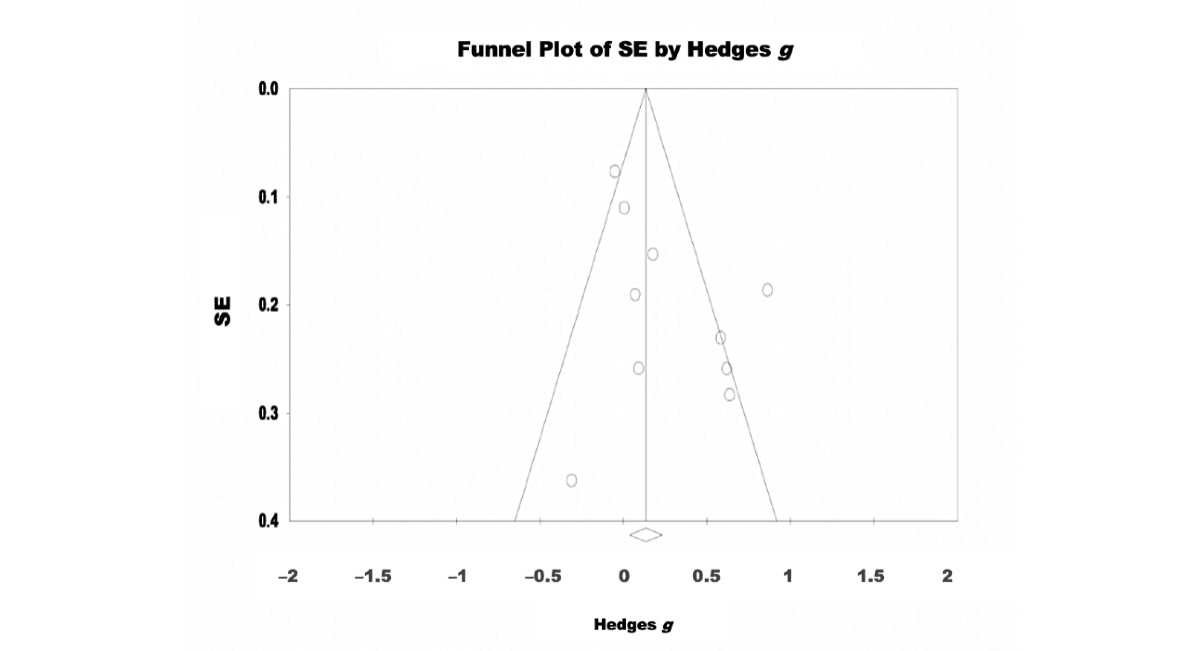
Anxiety–funnel plot [57,60,63].

A random-effects model was used to analyze the 59% (10/17) of the studies that assessed the effectiveness of eHealth interventions for the treatment of anxiety symptoms. There were 1668 participants included in total (intervention: 816, 48.92% and control: 852, 51.08%). The pooled effect size reflected a significant effect of eHealth interventions on anxiety symptoms with a small effect size (Hedges *g*=0.262, 95% CI 0.046-0.478; Z=2.379; *P=*.02; Figure 4). Significant heterogeneity was observed among the studies (Q=34.103; *P*<.001; *I*^2^=73.609). The test of asymmetry funnel plot is displayed in Figure 5. Egger regression test found no evidence of publication bias among the studies (b=2.33; *t*_8_=1.98; SE 1.18; *P*=.08). The Begg and Mazumdar rank correlation was nonsignificant (Kendall *S* statistic=9; *Τ*=0.200; Z=0.805; *P*=.42).

#### Sensitivity Analyses

After systematically removing one study at a time, it was observed that all studies affected the meta-estimate of the effect size of eHealth interventions during pregnancy by >5%. Of the 10 studies, 6 (60%) studies affected the meta-estimate such that their removal made the estimate larger, where a significant association was noted when individual studies were excluded (*P=*.01-.03) [51,52,58,62,64,66]. Of the 10 studies, 3 (30%) studies affected the meta-estimate such that their (individual) removal made the estimate smaller; however, the effect of the intervention remained significant (*P=*.04) [53,54,67]. Of the 10 studies, 1 (10%) study affected the meta-estimate such that its removal made the estimate smaller and nonsignificant [62].

### Effectiveness of eHealth Interventions for Treatment of Insomnia Symptoms During Pregnancy

#### Overview

**Figure 6:**
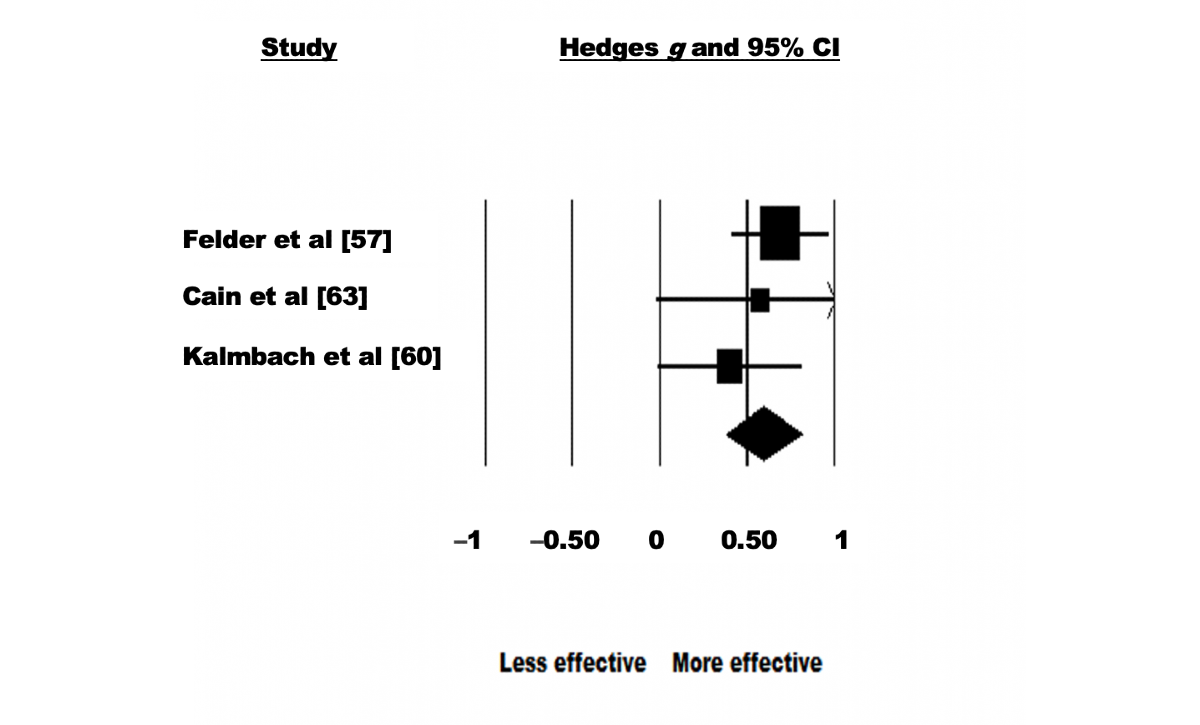
Insomnia–forest plot.

**Figure 7:**
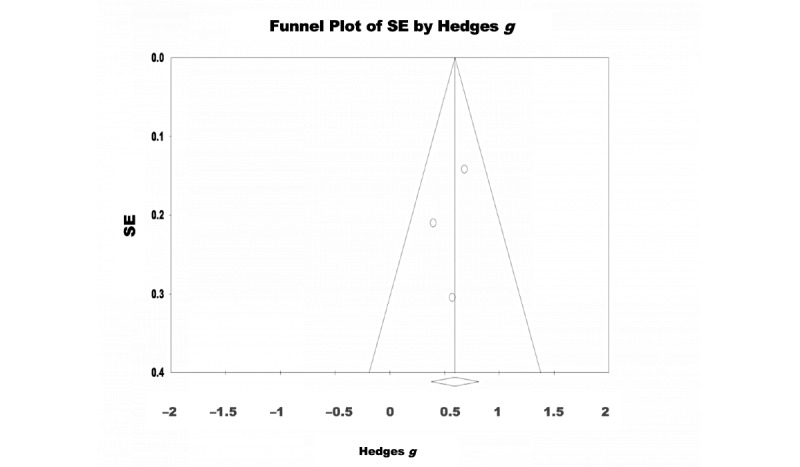
Insomnia–funnel plot.

A random-effects model was used to analyze the 18% (3/17) of the studies that assessed the effectiveness of eHealth interventions for the treatment of insomnia symptoms. There were 343 participants in total (intervention: 174, 50.7% and control: 169, 49.3%). The pooled effect size showed a significant effect of eHealth interventions on insomnia symptoms with a moderate effect size (Hedges *g*=0.595, 95% CI 0.379-0.811; Z=5.406; *P*<.001; Figure 6). No significant heterogeneity was observed among the studies (Q=1.259; *P*=.53; *I*^2^<0.001). The test of asymmetry funnel plot is displayed in Figure 7. Egger regression test found no evidence of publication bias among the studies (b=–1.27; *t*_1_=0.709; SE 1.80; *P=*.61). The Begg and Mazumdar rank correlation was nonsignificant (Kendall *S* statistic=–1; *Τ*=–0.33; Z=0.522; *P*=.60).

### Moderator Analyses

Using a meta-regression analysis, variables such as human monitoring (yes or no), risk of bias, type of control group used (active control vs nonactive control), treatment goal (treatment or prevention), baseline mental health symptoms (above or below), number of sessions, level of interactivity (web-based or hybrid), type of intervention (ie, CBT and mindfulness), structure (guided vs unguided), frequency (low or high), provider type (health care provider, researcher, or other), and eHealth type (internet or computer, text, and app) were noted as possible moderator variables for the observed effect sizes for both depressive and anxiety symptoms. Moderator analyses were not conducted for insomnia symptoms as there were not enough studies included to run the meta-regression. No significant moderators were detected for anxiety outcomes. However, for depressive outcomes, the meta-regression revealed that intervention type (mindfulness) significantly moderated depressive symptoms, where mindfulness interventions lead to better treatment outcomes in comparison with other intervention types (Tables 6 and 7).

**Table 6:** Moderators of eHealth intervention effectiveness on depressive symptoms using meta-regression analyses.

Moderator			Depression measure												
			N		β		SE		95% CI		Z value		Q		*P* value
Human monitoring^a^			11		.317		0.201		–0.077 to 0.711		1.58		2.49		.12
Risk of bias			11		–.023		0.131		–0.280 to –0.234		–0.18		0.03		.86
Type of control group^b^			11		–.254		0.248		–0.741 to 0.232		–1.02		1.05		.31
Treatment goal^c^			11		.104		0.234		–0.358 to 0.566		0.44		0.20		.66
Baseline symptoms^d^			11		–.018		0.228		–0.465 to 0.429		–0.08		0.01		.94
Number of sessions			9		–.006		0.010		–0.026 to 0.014		–0.57		0.33		.57
Interactivity (web-based or hybrid)^e^			11		.247		0.205		–0.156 to 0.649		1.20		1.44		.23
Guided versus unguided^f^			11		–.089		0.233		–0.544 to 0.367		–0.38		0.15		.70
**Intervention type^g^**															
	ABMT^h^	11		–.0124		0.405		–0.805 to 0.781		–0.03		9.86		.98	
	CBT^i^	11		.4065		0.229		–0.042 to 0.855		1.78		9.86		.08	
	Mindfulness	11		.510		0.176		0.166 to 0.854		1.91		9.86		.004	
Frequency^j^			11		.339		0.319		–0.286 to 0.964		1.06		1.13		.39
**Type of provider^k^**															
	Health care provider	11		–.030		0.250		–0.520 to 0.460		–0.12		1.21		.90	
	Researcher	11		.464		0.438		–0.394 to 1.322		1.06		1.21		.29	
**eHealth type^l^**															
	App	11		.169		0.206		–0.235 to 0.573		0.82		0.67		.41	

^a^No human monitoring was used as the reference group.

^b^Nonactive control group was used as the reference group.

^c^Prevention was used as the reference group.

^d^Below clinical cutoff was used as the reference group.

^e^Web-based interactivity was used as the reference group.

^f^Unguided was used as the reference group.

^g^Psychoeducation was used as the reference group.

^h^ABMT: attention bias modification training.

^i^CBT: cognitive behavioral therapy.

^j^Low frequency was used as the reference group.

^k^*Other* was used as the reference group.

^l^Internet was used as the reference group.

**Table 7:** Moderators of eHealth intervention effectiveness on anxiety symptoms using meta-regression analyses.

Moderator		Anxiety measure						
		N	β	SE	95% CI	Z Value	Q	*P* value
Human monitoring^a^		10	.110	0.238	–0.357 to 0.577	0.46	0.21	.64
Risk of bias		10	.034	0.119	–0.200 to 0.267	0.28	0.08	.78
Type of control group^b^		10	–.262	0.262	–0.776 to 0.252	–1	1.00	.32
Treatment goal^c^		10	.142	0.212	–0.275 to 0.558	0.67	0.44	.51
Baseline symptoms^d^		10	.0645	0.289	–0.501 to 0.630	0.22	0.05	.82
Number of sessions		8	.017	0.020	–0.022 to 0.055	0.84	0.71	.40
Interactivity (web-based or hybrid)^e^		10	–.073	0.240	–0.543 to 0.398	–0.30	0.09	.76
Guided versus unguided^f^		10	–.213	0.258	–0.719 to 0.294	–0.82	0.68	.41
**Intervention type^g^**								
	ABMT^h^	10	–.311	0.504	–1.299 to 0.677	–0.62	5.06	.54
	CBT^i^	10	.349	0.339	–0.315 to 1.012	1.03	5.06	.30
	Mindfulness	10	.534	0.293	–0.041 to 1.109	1.82	5.06	.07
General health		10	.233	0.318	–0.386 to 0.861	0.75	5.06	.46
Frequency^j^		10	.295	0.284	–0.263 to 0.852	1.04	1.08	.30
**Type of provider^k^**								
	Health care provider	10	–.370	0.263	–0.885 to 0.145	–1.41	2.11	.16
	Researcher	10	–.225	0.254	–0.722 to 0.272	–0.89	2.11	.38
**eHealth type^l^**								
	Smartphone app	10	–.041	0.256	–0.543 to 0.459	–0.16	0.84	.87
	SMS text message	10	.372	0.449	–0.508 to 1.252	0.83	0.84	.41

^a^No human monitoring was used as the reference group.

^b^Nonactive control group was used as the reference group.

^c^Prevention was used as the reference group.

^d^Below clinical cutoff was used as the reference group.

^e^Web-based interactivity was used as the reference group.

^f^Unguided was used as the reference group.

^g^Psychoeducation was used as the reference group.

^h^ABMT: attention bias modification training.

^i^CBT: cognitive behavioral therapy.

^j^Low frequency was used as the reference group.

^k^*Other* was used as the reference group.

^l^Internet was used as the reference group.

## Discussion

### Principal Findings

This systematic review and meta-analysis found that eHealth interventions reduced symptoms of anxiety and depression during pregnancy; however, the effect sizes for the treatment of depression and anxiety symptoms were small. eHealth, specifically CBT-I, was associated with improved insomnia symptoms during pregnancy, with a moderate effect size. None of the moderators of treatment response that we investigated emerged as significant, with the exception of intervention type being a significant moderator for depressive outcomes.

Findings showing a small effect size across eHealth interventions are consistent with the findings of other meta-analyses in this area. For instance, a study on the effectiveness of computer-based CBT for the treatment of depressive and anxiety symptoms in adolescent populations reported small to moderate effect sizes [68]. Another meta-analysis that investigated the efficacy of smartphone-based mental health interventions [69] found a small positive effect for individuals within the general population with depressive symptoms. In a meta-analysis that observed eHealth interventions for depression and anxiety in the general population, the overall effect size between intervention and control for depression and anxiety outcomes was small [42]. The consistent findings of small effect sizes for eHealth interventions targeting anxiety and depressive symptoms highlight the need for additional modifications that could increase effectiveness.

The small effect sizes observed for the use of eHealth interventions may be owing to the lack of factors theorized to improve program impact, including human monitoring, mood feedback, and high dropout rates in eHealth studies [70]. In this study, only 29% (5/17) of the studies included used human monitoring [51,52,63,65,66]. Furthermore, studies reported moderate to high attrition rates, which were as high as 75% [59]. As noted during the sensitivity analysis for the anxiety meta-estimate, the removal of the study by Chan et al [62] made the meta-estimate nonsignificant. Part of what may differentiate the study by Chan et al [62] from the other studies included in this meta-analysis is that participants were sent multiple prompts via email if it was noticed that they were not logging into the app. Another helpful component noted in the study by Chan et al [62] was that participants could directly message their obstetrics and gynecology physicians for any questions that they may have—again highlighting the importance of reminders and human monitoring throughout eHealth interventions.

There was also variability in the type of control groups used across the included studies, where some studies used a TAU control group, some used a waitlist control group, and others used an *active* control group (where control participants receive some sort of intervention that differs from the actual treatment). For example, in some studies, participants assigned to the control group were provided psychoeducation [61,63]. Although the use of an active control tends to reduce the observed effect sizes, moderator analyses found that the type of comparison group did not moderate the effectiveness of eHealth interventions. Furthermore, it should be noted that most studies included in the review used control conditions that were *TAU* and comparing with TAU may inflate effect sizes, as any sort of intervention is expected to be more helpful than no intervention. eHealth interventions do not necessarily need to outperform pre-existing face-to-face visits for them to be implemented in regular practice and these findings show that they likely outperform TAU.

For depression, the type of intervention emerged as a significant moderator of treatment effect (b=0.510; *P=*.004). Specifically, studies using mindfulness eHealth interventions had significant treatment outcomes compared with other interventions including CBT, attention bias modification training, and psychoeducation. Other intervention types did not significantly moderate the treatment effect, though CBT was approaching significance (b=0.407; *P=*.08). This finding could suggest that mindfulness eHealth interventions are more effective than CBT eHealth interventions under certain circumstances. For example, a study comparing CBT and mindfulness-based cognitive therapy to treat anxiety and depression in a diabetic population found that individuals with higher educational attainment responded better to mindfulness-based cognitive therapy compared with CBT [71].

Regarding anxiety, results from the moderator analyses showed that none of the hypothesized moderators influenced the effectiveness of the eHealth intervention on anxiety symptoms. Potentially, the null results in this study may be owing to the small number of studies included, thus limiting the statistical power of the study. However, the overall lack of significant moderators in this review is similar to results from recent meta-analyses, which also found that risk of bias, number of sessions, and therapist guidance (guided or unguided) did not significantly moderate depression and anxiety outcomes in eHealth intervention [72]. In contrast, findings from other meta-analyses on eHealth interventions in the general population suggest that intervention-level variables such as whether the intervention was guided or unguided, number of sessions, and the type of comparison group chosen significantly influenced the outcome [73,74]. Future researchers should continue to examine the possibility of moderators that were not investigated in this review, which may moderate treatment outcomes. For instance, experiences of poverty, racism, medical system marginalization, and single parent status are all moderators, which are known to contribute to elevated risk for maternal–child outcomes [75,76]. For example, in a study by Giscombé and Lobel [76] the authors found that compared with European Americans, African American infants show disproportionately higher rates of low birth weight, preterm delivery, and death during the first year of life. The review of the literature reveals that these outcomes are explained partly by various factors including socioeconomic status, higher levels of stress in African American women, racism, and ethnic differences in certain stress-related processes [76]. Similarly, another review revealed higher odds of low birth weight, preterm birth, stillbirth, and infant mortality among various indigenous populations [77]. Consequently, these various types of experiences can have a negative impact on treatment outcome. As such, these experiences should be taken into consideration when generalizing treatment efficacy to a heterogeneous population of women of various racial and socioeconomic backgrounds.

The eHealth intervention treatment of antenatal insomnia produced the largest effect size. However, this finding should be interpreted with caution, because only 3 studies on insomnia were included in this analysis. However, all of the included insomnia studies were evidence-based psychological interventions and this finding is in line with the general CBT-I literature, which has shown moderate to large effect sizes when treatment is delivered digitally [78]. The moderate effect sizes observed in eHealth interventions treating insomnia in comparison with the small effect sizes for eHealth interventions treating anxiety and depression may be owing to the use of standardized and highly behavioral treatment protocols that are established to work in person. Presentation of anxiety and depression may arise owing to myriad factors (ie, genetics, work, social support, partner support, and socioeconomic status), some of which may not be adequately targeted through digital intervention.

### Limitations

These findings should be interpreted in the context of several limitations. First, the sample sizes of the selected studies were small. In addition, most of the studies had moderate to high levels of participant attrition—a problem that is commonly identified in the eHealth literature [79]. High levels of attrition are attributed to the lack of human interactions in some eHealth interventions [80]. There was also high heterogeneity among interventions treating symptoms of anxiety and depression. Methodological variations within selected studies included variability in intervention, intervention intensity, duration of the intervention, and mode of eHealth delivery (ie, app, SMS text message, and internet). It should be noted that although telephone-based studies were also eHealth interventions of interest, the review did not identify any telephone-based studies, which may be owing to the rise in technology, which is supported by the fact that most of the interventions were delivered through the internet (12/17, 71%). Another limitation of this research is that most of the studies included in the review were conducted in the context of a high-income country rather than low-income countries, which may limit the generalizability of the findings. Fathers have significantly elevated rates of depression and anxiety during pregnancy and the postpartum period [81,82]; however, this meta-analysis found no studies that focused on men and partners during pregnancy, despite this having been an initial goal in the search. Research concerning fathers and partners during pregnancy is a future direction for eHealth research. Participants’ race and ethnicity were also rarely reported in the included studies, limiting our ability to examine moderation by race and ethnicity. This is a common limitation of RCTs and should be addressed in future studies [83,84].

### Future Directions

The accessibility to rural or underserved areas and flexibility of eHealth interventions makes eHealth an important part of health care beyond the pandemic. Given that eHealth interventions can take the form of web-based programs, remote monitoring, teleconsultation, and mobile device–supported programs, eHealth interventions provide many potential avenues for pregnant women to receive care for mental health problems. Future eHealth trials should consider a stepped model for mental health interventions [85-87], whereby mild mental health symptoms can be matched with lower-resourced interventions, such as eHealth. More significant symptoms are matched with face-to-face intervention, longer sessions, and more clinician interaction [87]. eHealth could also be used to track patient symptoms, which could be a promising way to detect worsening mental health and prevent future symptom deterioration. In addition, partners and fathers are also subject to symptoms of anxiety and depression perinatally. As such, during pregnancy, fathers and partners may benefit from adapted eHealth treatments [81].

eHealth intervention trials should also consider implementing conditions that have asynchronous activities (ie, video modules) compared with synchronous activities (intensive guidance via the web) [86]. Comparison of these modes of delivery would indicate which mode successfully implements adherence to the app or intervention. This could inform future interventions for methods to decrease attrition and increase engagement.

In addition, further emphasis should be placed on understanding the potential that eHealth interventions may have for communities that have faced medical marginalization and maternal–child health disparities. It is important to consider that health disparities and inequities are often the result of adverse social determinants (including issues related to service access, racism, colonialism, and stigmatization) [88]. For example, research has found that indigenous women do not seek help owing to stigma, racism, fear of being blamed or labeled as a bad parent, and for fear of child apprehension [89]. As such, future research can investigate how eHealth interventions can be tailored to be more culturally sensitive, and thus, more appealing to marginalized groups.

Moreover, it should be noted that not everyone in Canada and other parts of the world have access to the means necessary to engage in eHealth interventions. For example, not everyone has reliable internet or access to computers, telephones, or smartphones. Therefore, health care systems should consider how to provide better support for infrastructure and equipment, specifically for those in rural, remote, and indigenous communities. For instance, only 24% of First Nation reserves in Canada have access to reliable internet [90]. Furthermore, in the United States, low-income Hispanics are the most digitally underserved population and this is not owing to a lack of interest in using internet health services [91]. Rather, this finding stems from barriers including low income, poor digital competence, and limited English proficiency [91]. Furthermore, often, devices are also shared among family members, which also limits internet access. Thus, research should focus on determining how eHealth interventions can be streamlined into the current health care system to increase accessibility to mental health services.

In addition, the finding that none of the included studies required a formal diagnosis of anxiety, depression, or insomnia at baseline is also common in the larger literature of nonspecialist-delivered interventions in high-income countries. This finding may be related to some of the barriers in the delivery of psychological treatment, which include the lack of skilled providers who can provide a formal diagnosis [92]. However, this barrier may be overcome if current trends in global mental health move toward transdiagnostic approaches, which focus on common elements of mental health rather than focusing on any 1 specific disorder [92]. This is because recent evidence suggests that targeting *common elements* including behavioral activation, communication, and problem solving can reduce the complexity of needing to learn diverse psychological treatment packages for specific clinical phenotypes (such as depression, anxiety, and stress‐related disorders) [92]. Moreover, we see that most of the included studies had interventions that were facilitated by clinicians and peers. However, recent research suggests that a potential solution to increasing accessibility of mental health services is to use nonspecialist providers (ie, nurse practitioners and nurses) and train them in the delivery of brief and low-intensity interventions, which has consistently been found to have moderate to strong effects in reducing distress associated with mental health concerns [23].

Finally, future research on eHealth can be directed toward determining how eHealth interventions can be smoothly integrated into the current health care system to streamline patient care and increase patient accessibility to mental health services.

### Conclusions

In conclusion, this review demonstrated that eHealth interventions reduced symptoms of anxiety, depression, and insomnia in individuals during pregnancy in comparison with controls. eHealth interventions for anxiety, depression, and insomnia symptoms hold promise as adjuncts to other clinical approaches and as a component to stepped-care models of treatment for mental health problems [93,94].

## Appendix

Multimedia Appendix 1 Search strategy used for the review.

## Abbreviations

CBT: cognitive behavioral therapy
CBT-I: cognitive behavioral therapy for insomnia
PRISMA: Preferred Reporting Items for Systematic Review and Meta-Analysis
PROSPERO: International Prospective Register of Systematic Reviews
RCT: randomized controlled trial
TAU: treatment as usual
