# Supplementary figures and images for: eHealth Interventions for Treatment and Prevention of Depression, Anxiety, and Insomnia During Pregnancy: Systematic Review and Meta-analysis
Source: JMIR Ment Health. 2022 Feb 21;9(2):e31116. doi: 10.2196/31116 (PMC8902665; doi:10.2196/31116)

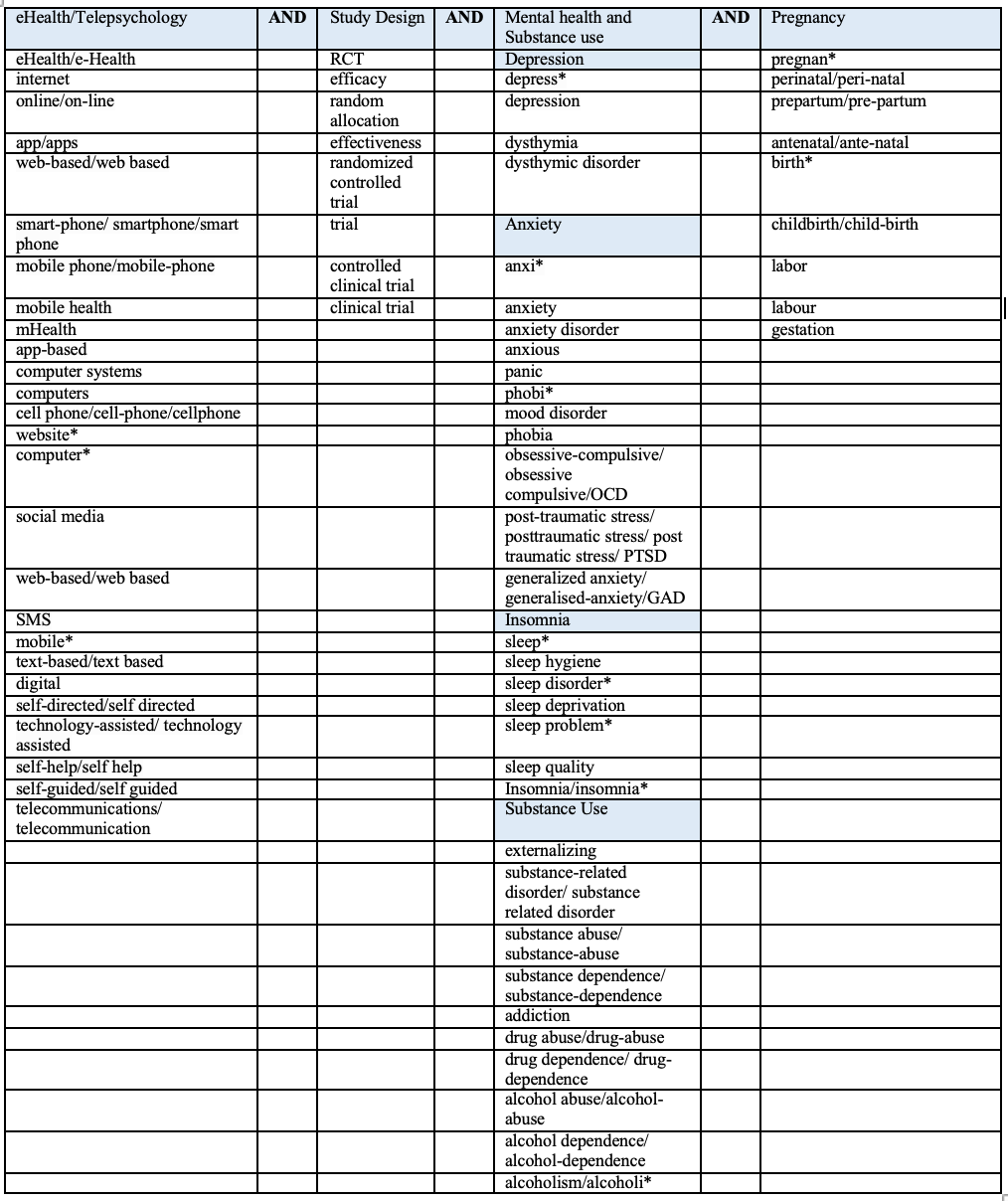

Supplement: Multimedia Appendix 1 [file mental_v9i2e31116_app1.png]
